# Supplementary material for: The Investigation of Somatostatin Receptors as a Potential Target in Breast Phyllodes Tumours
Source: Diagnostics (Basel). 2024 Dec 17;14(24):2841. doi: 10.3390/diagnostics14242841 (PMC11675630; doi:10.3390/diagnostics14242841)
Supplement: Supplementary file 1 [file diagnostics-14-02841-s001.zip › diagnostics-3278466-supplementary.pdf]

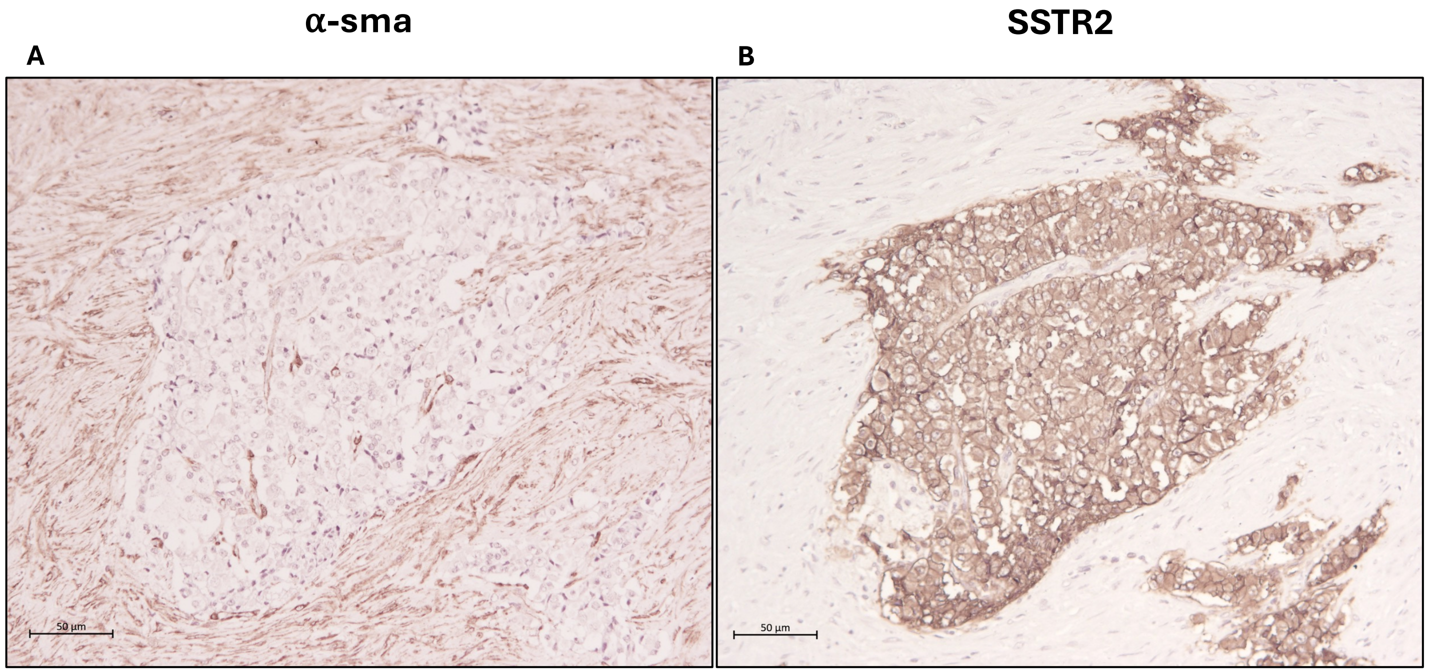

Supplementary Figure S1: In PanNET tumours SSTR2 expression is confined to epithelial cells. α-sma-positive cells do not express SSTR2.

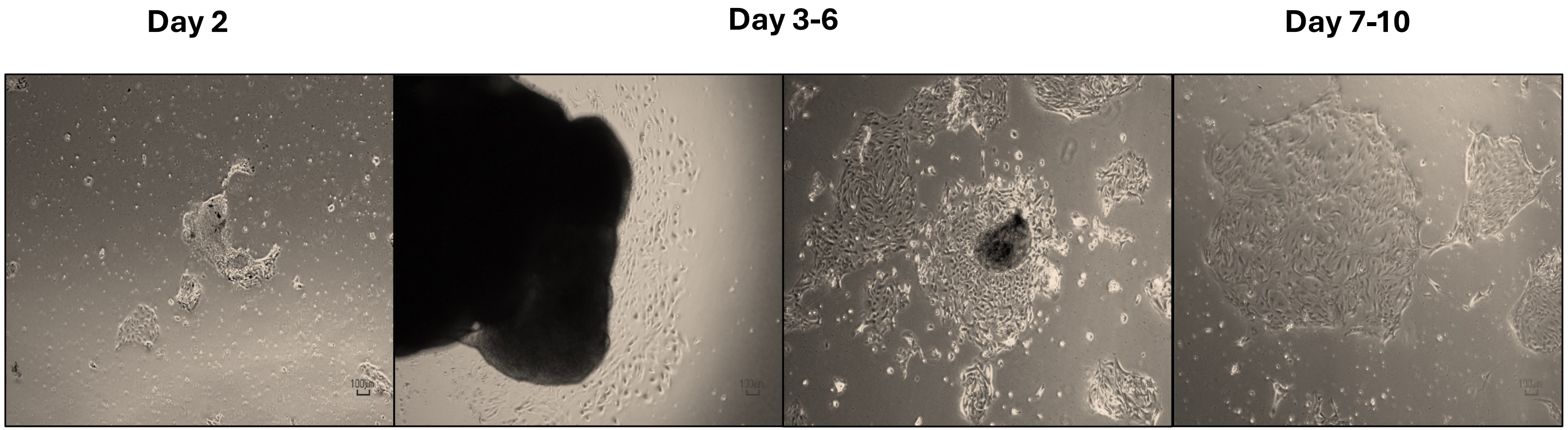

Supplementary Figure S2: Cells outgrown from the explants constituting a heterogenous cell population containing epithelial-like cobblestone-shaped cells and spindle-shaped mesenchymal cells.
